# Insecticide resistance to insect growth regulators, avermectins, spinosyns and diamides in *Culex quinquefasciatus* in Saudi Arabia

**DOI:** 10.1186/s13071-021-05068-8

**Published:** 2021-10-29

**Authors:** Abdulwahab M. Hafez, Naeem Abbas

**Affiliations:** Pesticides and Environmental Toxicology Laboratory, Department of Plant Protection, College of Food and Agriculture Sciences, King Saud University, P.O. Box 2460, Riyadh, 11451 Saudi Arabia

**Keywords:** Mosquitoes, Field-evolved-resistance, Diflubenzuron, Chlorantraniliprole, Biopesticides, Insect vector management

## Abstract

**Background:**

*Culex quinquefasciatus* is not only a biting nuisance but also an important vector of fatal diseases. In Saudi Arabia, management measures to control this mosquito and thereby prevent associated disease transmission have focused on insecticides. Nevertheless, information on the resistance status of *C. quinquefasciatus* is insufficient, especially concerning insecticides containing novel classes of insecticides.

**Methods:**

We evaluated six insecticides belonging to four classes of insecticides (insect growth regulators [3], avermectins [1], diamides [1] and spinosyns [1]) for toxicity and resistance in eight *C. quinquefasciatus* populations (from Ishbiliya, Al-Masfa, Al-Masanie, Al-Washlah, Al-Nakhil, Irqah, Al-Suwaidi and Al-Ghanemiya) following World Health Organisation protocols.

**Results:**

Resistance status ranging from susceptibility/low resistance to high resistance, in comparison with the susceptible strain, was detected for cyromazine in the eight *C. quinquefasciatus* populations: Ishbiliya (resistance ratio [RR] = 3.33), Al-Masfa (RR = 4.33), Al-Masanie (RR = 3.67), Al-Washlah (RR = 2.33), Al-Nakhil (RR = 5.33), Irqah (RR = 7.00), Al-Suwaidi (RR = 21.33) and Al-Ghanemiya (RR = 16.00). All *C. quinquefasciatus* populations exhibited a high level of resistance to diflubenzuron (RR = 13.33–43.33), with the exception of Al-Nakhil which exhibited moderate resistance (RR = 10.00). Susceptibility/low resistance to high resistance was observed for triflumuron in the eight *C. quinquefasciatus* populations: Ishbiliya (RR = 0.50), Al-Ghanemiya (RR =  3.00), Al-Suwaidi (RR =  10.00), Al-Masfa (RR =  5.00), Al-Masanie (RR =  10.00), Al-Nakhil (RR =  5.00), Irqah (RR =  5.00) and Al-Washlah (RR =  15.00). Susceptibility/low resistance was assessed for abamectin, chlorantraniliprole and spinosad in all *C. quinquefasciatus* populations, with RR ranges of 0.25–3.50, 0.17–2.19, and 0.02–0.50, respectively. However, the population collected from Irqah showed high resistance to chlorantraniliprole (RR = 11.93).

**Conclusions:**

The detection of widespread resistance to insect growth regulators in *C. quinquefasciatus* highlights an urgent need to establish integrated vector management strategies. Our results may facilitate the selection of potent insecticides for integrated vector management programmes for *C. quinquefasciatus*.

**Graphical Abstract:**

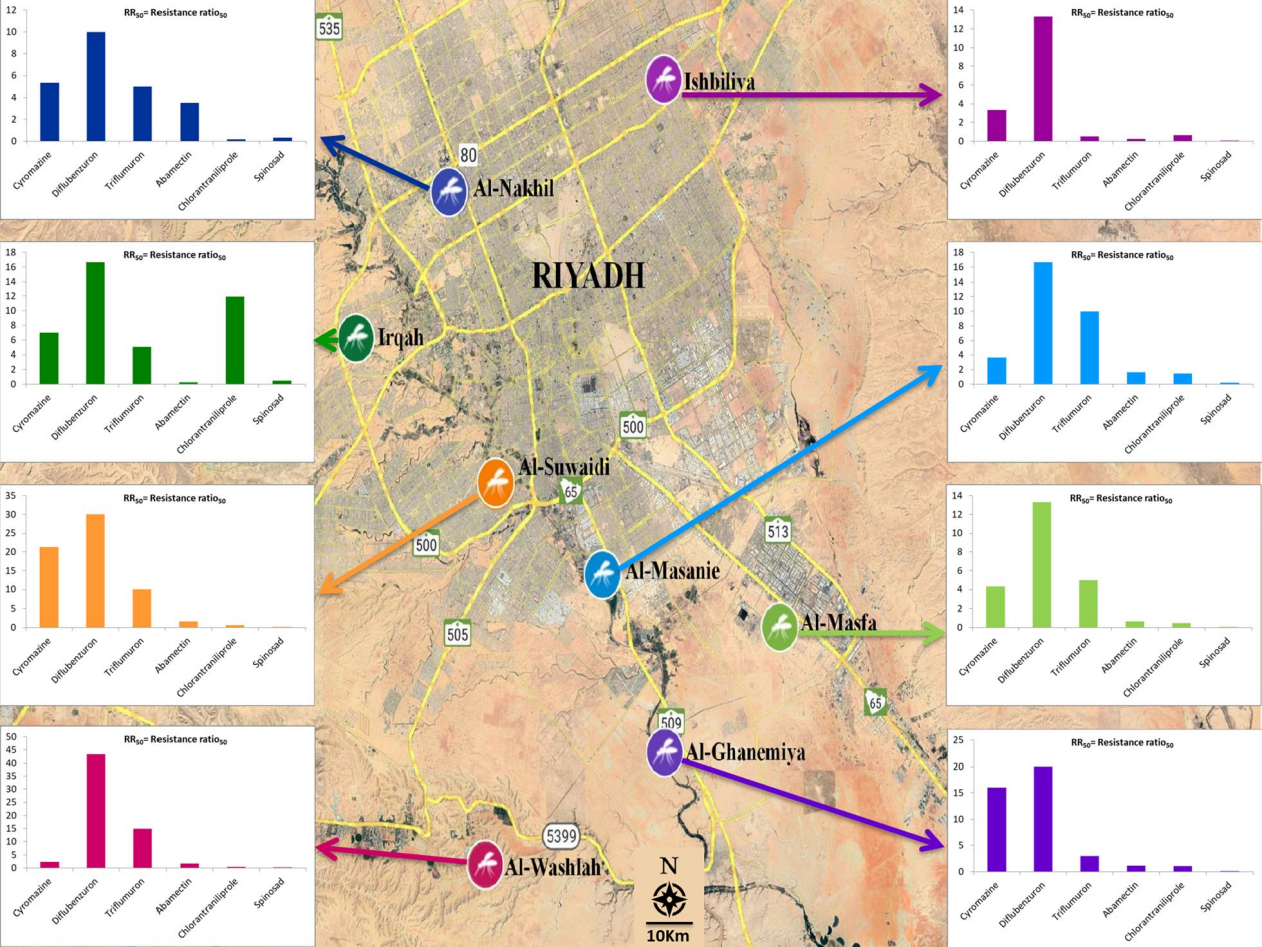

## Background

*Culex quinquefasciatus* is not only a biting nuisance to its hosts [1, 2] but also a significant insect vector of various parasites, including West Nile fever, lymphatic filariasis and Japanese encephalitis [3–5]. *Culex quinquefasciatus* can breed in various aquatic habitats, including mangrove swamps, fresh or salt water marshes, stream and river edges and temporary standing rain water [6–8], and human settlements, agricultural lands and livestock farms with inadequate hygienic practices/facilities are also ideal breeding habitats.

Management measures, such as the use of genetic manipulation, sterile insect techniques, eradication of breeding places, chemical insecticides and natural biological agents, have been used to control insect vectors, including *C*. *quinquefasciatus*, worldwide [1, 9, 10]. Of these, the management of *C*. *quinquefasciatus* to prevent associated parasite transmission has focused on insecticides as a rapid control measure [11, 12]. At the present time, avermectins, diamides and spinosyns, are being used widely to control the larvae of *C*. *quinquefasciatus* and other insect vectors worldwide [1, 13, 14]. However, the extensive and widespread use of these insecticides has led to resistance in *C*. *quinquefasciatus* in different parts of the world [1, 15–19] and, in adddition, has caused environmental pollution, increases in the preventive costs of chemical control and destroyed nontarget organisms [20–22]. Taken together, these factors necessitate the use of integrated vector management programmes against *C*. *quinquefasciatus*.

The IGRs cyromazine, diflubenzuron and triflumuron are currently the most effective larvicides for controlling mainly dipteran pests, including mosquitoes [13, 23–25]. Cyromazine is a molting disruptor, whereas diflubenzuron and triflumuron are chitin synthesis inhibitors. Abamectin (an avermectin) and spinosad (a spinosyn) are biorational insecticides, with the former being a glutamate-gated chloride channel allosteric modulator and the latter a nicotinic acetylcholine receptor allosteric modulator [26]. Chlorantraniliprole is an anthranilic diamide insecticide; it acts as a ryanodine receptor modulator in insect muscles [26], causing the uncontrolled release of calcium ions that leads to feeding cessation, lethargy, muscle paralysis and ultimately death [27, 28]. Because of their low mammalian toxicity and low hazard threat to the target’s natural enemies, these insecticides are good candidates for the management of various insect pests, including mosquitoes [29–31].

**Fig. 1:**
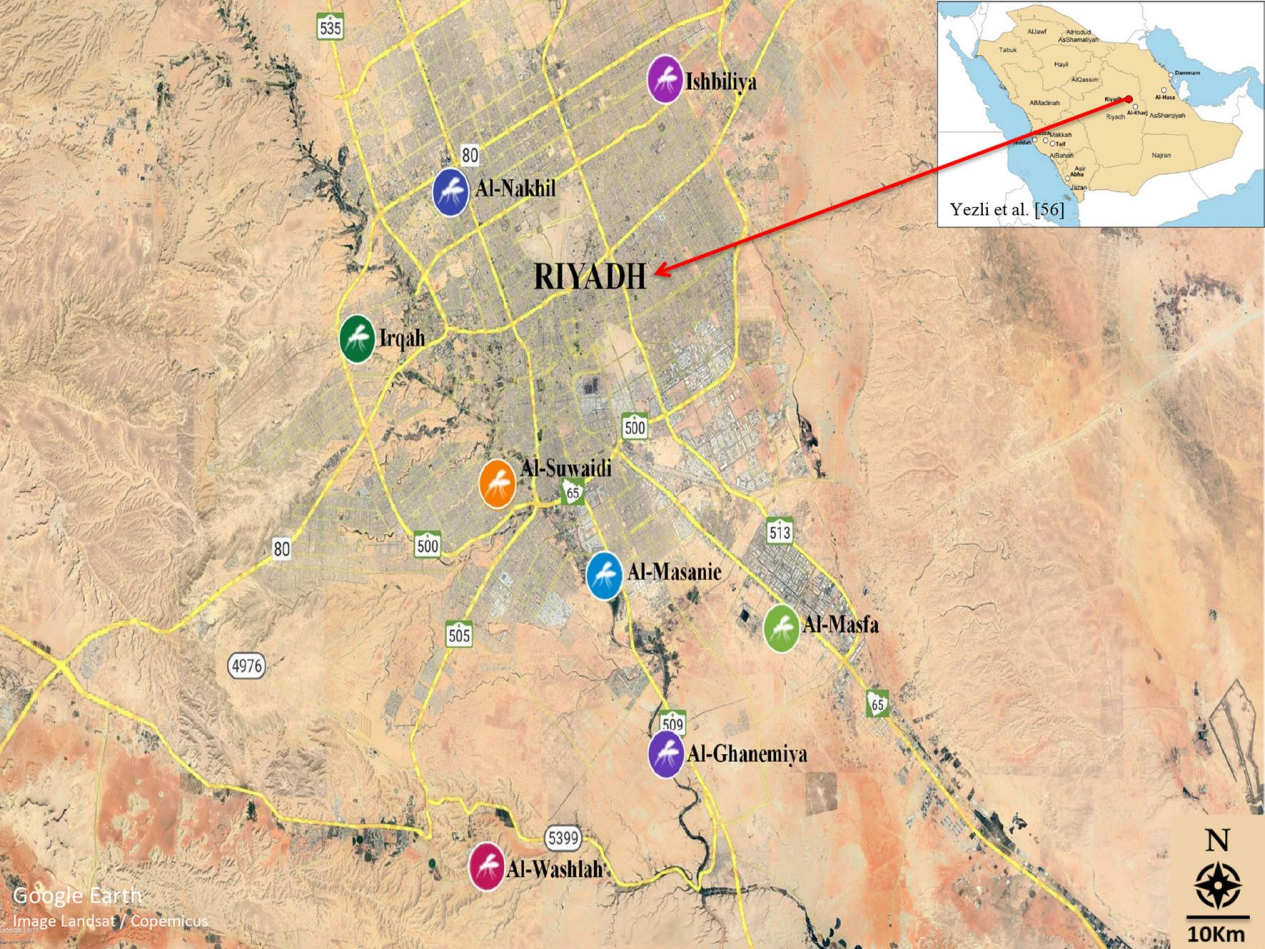
Location of sites where* Culex quinquefasciatus* field populations were collected in Riyadh city. See Table 1 for abbreviations of study/population collection sites

Mosquito control programmes in Riyadh, Saudi Arabia rely mainly on chemical control. Consequently, there is always the possibility of the development of insecticide resistance, which would, for example, reduce the effectiveness of chemical control against *C*. *quinquefasciatus*. It is crucial to know the resistance status of *C*. *quinquefasciatus* to newly developed insecticides before their widespread use in Riyadh (Fig. 1). Hence, we evaluated the toxicity and resistance levels of six novel insecticides in the IGR, avermectin, diamide, and spinosyn classes against eight *C*. *quinquefasciatus* larval populations from different areas in Riyadh, Saudi Arabia. Baseline susceptibility data from this study will help in the design of appropriate and effective strategies for controlling *C*. *quinquefasciatus*.

## Methods

### *Culex quinquefasciatus* populations

Approximately 200 *C. quinquefasciatus* larvae at mixed developmental stages were collected from standing water or from containers at each of the eight different study locations in the environs of Riyadh, Saudi Arabia; these sites were designated ISH, SUW, GHN, MSF, MSN, NKL, WSH and IRQ (Fig. 1; Table 1). The locations were selected on the basis that there was a high chance they had been exposed to pesticides. Each population was collected and maintained separately. After collection, larvae of each population were transferred to alternative plastic containers (4 l) in the laboratory and provided cattle food ad libitum for feeding until pupation. The emerged adults were transferred into cages (30 × 30 cm), and cotton wicks soaked in 10% sugar solution were provided as food. The cotton wicks were moistened every 2 days and replaced when they became dirty. A restrained pigeon with keel feathers removed was provided for 12 h overnight for blood-feeding during each oviposition period. Plastic cups (500 ml) containing tap water were placed in the adult cages to receive egg masses and to obtain uniform F_1_ populations. The plastic cups were then removed and the eggs transferred into plastic containers. Hatched larvae were provided with cattle food ad libitum and third-instar larvae were used for the bioassay. All populations were maintained separately in the laboratory at 27℃ ± 2 °C, 65% ± 5% humidity and under a 12:12 h (light: dark) photoperiod.

**Table 1:** History of *Culex quinquefasciatus* field populations collected from Riyadh city

Population	Location	Coordinates	Collection month, year	Number of larvae (approximate estimate)
ISH	Ishbiliya	24.802°N, 46.803°E	January, 2020	200
SUW	Al-Suwaidi	24.590°N, 46.676°E	January, 2020	200
GHN	Al-Ghanemiya	24.482°N, 46.798°E	January, 2020	200
MSF	Al-Masfa	24.471°N, 46.861°E	January, 2020	200
MSN	Al-Masanie	24.558°N, 46.743°E	January, 2020	200
NKL	Al-Nakhil	24.737°N, 46.620°E	January, 2020	200
WSH	Al-Washlah	24.409°N, 46.660°E	January, 2020	200
IRQ	Irqah	24.677°N, 46.575°E	January, 2020	200

The susceptible reference strain (designated as SUS) was obtained from the High Institute of Public Health, Alexandria University, Egypt, in 1990 and has been maintained since then under the above-mentioned protocol with no exposure to any kind of chemicals.

### Insecticides

The following six commonly used commercial formulations of insecticides belonging to four classes were used for larval bioassays: (i) the IGRs cyromazine (Novasat 75WP; Astranova Chemicals, Antalya, Turkey), diflubenzuron (Diflon 250WP; Saudi Delta Company, Riyadh, Saudi Arabia) and triflumuron (Starycide 480SC; Bayer CropScience, St. Louis, MO, USA); (ii) the avermectin abamectin (Malactin 36EC; Shams Badeel Factory, Riyadh, Saudi Arabia); (iii) the diamide chlorantraniliprole (Coragen 20SC; FMC Corp., Philadelphia, PA, USA); and (iv) the spinosyn spinosad (Tracer 40SC; Dow AgroSciences Ltd., Abingdon, UK).

### Larva bioassays

The bioassays of the insecticides against *C. quinquefasciatus* larvae were performed following the protocol proposed by the World Health Organisation [32]. For each insecticide, five concentrations that caused mortality ranging from > 0% to < 100% were prepared in tap water by serial dilution from a stock solution (1000 ml). A fresh stock solution was prepared for each replication, and assays were performed at different times to ensure true replication [33]. Third-instar larvae from each pooled population were kept in plastic cups containing 400 ml of the test solution. Insecticide dilution bioassays were performed four times, with 10 larvae per replicate and a total of 240 larvae in each bioassay. A total of 40 larvae (four replicates/9 larvae in each) were used in the control treatment. All bioassays were conducted and maintained under the above-mentioned laboratory conditions. Mortality was recorded after 48 h for the abamectin, chlorantraniliprole and spinosad treatment groups. For the three IGR groups, mortality data were recorded after adult emergence, with pupae failing to emerge as adults considered to be dead.

### Data analyses

The bioassay data were analysed using POLO Plus software [34] to determine the median lethal concentration (LC_50_), 95% fiducial limits (FLs), standard error, and chi-squared (*χ*^2^) test. Using the Abbott [35] formula, mortalities were corrected when needed by reference to mortality in the control treatment. LC_50_ values were considered to be significantly different if their 95% FLs did not overlap [36]. Resistance ratios (RRs) were calculated as: LC_50_ for the field population/LC_50_ for the susceptible strain. The RRs were classified as follows: RR < 5 indicated susceptibility/low resistance; RR = 5–10 indicated moderate resistance and RR > 10 indicated high resistance [32, 37].

## Results

### Resistance to IGRs

The LC_50_ and RR values for the IGRs cyromazine, diflubenzuron and triflumuron for the eight *C. quinquefasciatus* field larval populations are reported in Table 2

**Table 2:** Resistance to insect growth regulators in *Culex quinquefasciatus* larva populations from Riyadh city

Insecticide	Population^a^	*n*	Concentration (µg/ml)	LC_50_ (95% FL) (mg/ml)	Slope ± SE	*χ*^2^	*df*	*P*	RR
Cyromazine	SUS	240	0.0010–0.0156	0.003 (0.002–0.004)	1.78 ± 0.25	0.66	3	0.88	1.00
	ISH	240	0.0039–0.0625	0.010 (0.008–0.013)	1.84 ± 0.26	3.27	3	0.35	3.33
	SUW	240	0.0039–0.0625	0.064 (0.037–0.222)	0.99 ± 0.24	0.73	3	0.87	21.33
	GHN	240	0.0039–0.0625	0.048 (0.024–0.476)	0.65 ± 0.22	0.38	3	0.94	16.00
	MSF	240	0.0039–0.0625	0.013 (0.008–0.019)	1.04 ± 0.22	0.68	3	0.88	4.33
	MSN	240	0.0039–0.0625	0.011 (0.005–0.018)	0.80 ± 0.22	0.61	3	0.89	3.67
	NKL	240	0.0039–0.0625	0.016 (0.012–0.022)	1.45 ± 0.23	0.95	3	0.81	5.33
	WSH	240	0.0039–0.0625	0.007 (0.001–0.015)	0.60 ± 0.21	0.13	3	0.99	2.33
	IRQ	240	0.0039–0.0625	0.021 (0.013–0.045)	0.80 ± 0.22	0.04	3	1.00	7.00
Diflubenzuron	SUS	240	0.0001–0.0020	0.0003 (0.0002–0.0004)	1.37 ± 0.23	0.99	3	0.80	1.00
	ISH	240	0.0020–0.0313	0.004 (0.003–0.005)	1.90 ± 0.27	5.56	3	0.14	13.33
	SUW	240	0.0039–0.0625	0.009 (0.007–0.011)	2.30 ± 0.30	3.08	3	0.38	30.00
	GHN	240	0.0039–0.0625	0.006 (0.004–0.008)	1.59 ± 0.26	1.62	3	0.65	20.00
	MSF	240	0.0039–0.0625	0.004 (0.002–0.007)	1.15 ± 0.25	1.49	3	0.68	13.33
	MSN	240	0.0039–0.0625	0.005 (0.002–0.007)	1.42 ± 0.26	1.50	3	0.68	16.67
	NKL	240	0.0039–0.0625	0.003 (0.001–0.006)	1.18 ± 0.25	3.38	3	0.34	10.00
	WSH	240	0.0039–0.0625	0.013 (0.010–0.017)	1.80 ± 0.25	0.34	3	0.95	43.33
	IRQ	240	0.0039–0.0625	0.005 (0.004–0.007)	2.28 ± 0.35	3.07	3	0.38	16.67
Triflumuron	SUS	240	0.00003–0.0005	0.0002 (0.0001–0.0002)	1.81 ± 0.33	1.76	3	0.62	1.00
	ISH	240	0.00002–0.0002	0.0001 (0.00004–0.0001)	1.90 ± 0.26	1.85	3	0.60	0.50
	SUW	240	0.0005–0.0078	0.002 (0.000–0.007)	0.51 ± 0.21	1.58	3	0.66	10.00
	GHN	240	0.0005–0.0078	0.0006 (0.0003–0.0009)	1.51 ± 0.29	4.56	3	0.21	3.00
	MSF	240	0.0005–0.0078	0.001 (0.000–0.001)	1.04 ± 0.24	0.12	3	0.99	5.00
	MSN	240	0.0005–0.0039	0.002 (0.001–0.003)	1.25 ± 0.31	1.08	2	0.78	10.00
	NKL	240	0.0005–0.0078	0.001 (0.001–0.002)	1.46 ± 0.24	4.21	3	0.24	5.00
	WSH	240	0.0005–0.0078	0.003 (0.002–0.004)	1.28 ± 0.23	2.29	3	0.51	15.00
	IRQ	240	0.0005–0.0078	0.001 (0.001–0.002)	0.78 ± 0.22	0.31	3	0.96	5.00

^a^See Table 1 for the full names of each study population/study location

FL, fiducial limits; LC_50_, median lethal concentration; *n*, number of larvae exposed in each bioassay; SE, standard error; RR, resistance ratio (LC_50_ of insecticide for the field population/LC_50_ of insecticide for the susceptible strain)

.

The LC_50_ for cyromazine ranged from 0.007 to 0.064 µg/ml. The populations ISH, MSF, MSN and WSH showed susceptibility/low resistance to cyromazine (RR = 3.33, 4.33, 3.67 and 2.33, respectively); two field populations, namely NKL and IRQ, showed moderate resistance (RR = 5.33 and 7.00, respectively) and the SUW and GHN field populations showed high resistance (RR = 21.33 and 16.00, respectively).

The LC_50_ values for diflubenzuron ranged from 0.003 to 0.013 µg/ml. All populations exhibited a high level of resistance to diflubenzuron (RR = 13.33–43.33), except for NKL, which exhibited moderate resistance (RR = 10.00).

The LC_50_ values for triflumuron ranged from 0.0001 to 0.003 µg/ml. The WSH population showed high resistance (RR = 15), the SUW, MSF, MSN, NKL and IRQ populations showed moderate resistance (RR = 10.00, 5.00, 10.00, 5.00 and 5.00, respectively) ISH and the GHN population showed susceptibility/low resistance (RR = 0.50 and 3.00, respectively).

### Resistance to abamectin, chlorantraniliprole and spinosad

The LC_50_ and RR values for abamectin, chlorantraniliprole and spinosad for the eight *C. quinquefasciatus* field larval populations are reported in Table 3.

**Table 3:** Resistance to abamectin, chlorantraniliprole and spinosad in *Culex quinquefasciatus* larval populations from Riyadh city

Insecticide	Population^a^	*n*	Concentration (µg/ml)	LC_50_ (95% FL) (mg/ml)	Slope ± SE	*χ*^2^	*df*	*P*	RR
Abamectin (avermectin class)	SUS	240	0.0020–0.0625	0.008 (0.006–0.010)	1.42 ± 0.19	1.83	4	0.77	1.00
	ISH	240	0.0010–0.0156	0.002 (0.001–0.004)	0.94 ± 0.21	0.47	3	0.93	0.25
	SUW	240	0.0020–0.0625	0.013 (0.009–0.020)	1.34 ± 0.21	2.98	4	0.56	1.63
	GHN	240	0.0020–0.0625	0.009 (0.007–0.012)	1.85 ± 0.24	3.76	4	0.44	1.13
	MSF	240	0.0039–0.0625	0.005 (0.002–0.007)	1.28 ± 0.29	0.50	3	0.92	0.63
	MSN	240	0.0020–0.0625	0.013 (0.008–0.021)	1.01 ± 0.20	1.70	4	0.79	1.63
	NKL	240	0.0039–0.0625	0.028 (0.015–0.099)	0.79 ± 0.25	0.20	3	0.98	3.50
	WSH	240	0.0039–0.0625	0.013 (0.009–0.018)	1.22 ± 0.23	1.05	3	0.79	1.63
	IRQ	240	0.0010–0.0156	0.002 (0.001–0.004)	0.78 ± 0.25	1.16	3	0.76	0.25
Chlorantraniliprole (diamide class)	SUS	240	0.0020–0.0625	0.029 (0.020–0.046)	1.26 ± 0.19	3.15	4	0.53	1.00
	ISH	240	0.0078–0.125	0.018 (0.000–0.051)	0.51 ± 0.24	0.43	3	0.93	0.62
	SUW	240	0.0020–0.0625	0.018 (0.014–0.024)	1.95 ± 0.26	0.87	4	0.93	0.62
	GHN	240	0.0020–0.0625	0.030 (0.020–0.055)	1.21 ± 0.22	3.81	4	0.43	1.03
	MSF	240	0.0020–0.0625	0.013 (0.008–0.021)	1.01 ± 0.20	0.11	4	0.99	0.45
	MSN	240	0.0020–0.0625	0.042 (0.024–0.121)	0.95 ± 0.21	0.44	4	0.98	1.45
	NKL	240	0.0039–0.0625	0.005 (0.000–0.011)	0.62 ± 0.25	0.62	3	0.89	0.17
	WSH	240	0.0039–0.0625	0.012 (0.009–0.015)	1.85 ± 0.27	1.52	3	0.68	0.41
	IRQ	240	0.2500–4.0000	0.346 (0.204–0.482)	1.54 ± 0.26	0.15	3	0.99	11.93
Spinosad (spinosyn class)	SUS	240	0.0001–0.0039	0.012 (0.004–0.511)	0.61 ± 0.18	0.66	4	0.96	1.00
	ISH	240	0.0020–0.0313	0.001 (0.000–0.003)	0.71 ± 0.21	0.58	3	0.90	0.08
	SUW	240	0.0001–0.0039	0.0003 (0.0002–0.0004)	2.25 ± 0.30	5.58	4	0.23	0.03
	GHN	240	0.0001–0.0039	0.0003 (0.0002–0.0004)	1.97 ± 0.28	1.15	4	0.89	0.03
	MSF	240	0.0001–0.0039	0.001 (0.0006–0.0014)	2.59 ± 0.53	4.82	4	0.31	0.08
	MSN	240	0.0020–0.0625	0.002 (0.002–0.003)	2.68 ± 0.50	1.38	4	0.85	0.17
	NKL	240	0.0002–0.0039	0.004 (0.003–0.009)	1.58 ± 0.33	2.27	3	0.52	0.33
	WSH	240	0.0002–0.0039	0.0002 (0.0001–0.0004)	1.10 ± 0.28	0.35	3	0.95	0.02
	IRQ	240	0.0020–0.0313	0.006 (0.003–0.010)	1.00 ± 0.26	1.11	3	0.77	0.50

^a^See Table 1 for the full names of each stud population/study location

The LC_50_ for abamectin ranged from 0.002 to 0.028 µg/ml, and the RRs ranged from 0.25 to 3.50 for all eight field populations, indicating that all populations were susceptible/showed low resistance to abamectin.

The LC_50_ for chlorantraniliprole ranged from 0.005 to 0.346 µg/ml and the RRs ranged from 0.17 to 11.93. Susceptibility/low resistance was observed in all field populations with the exception of the IRQ population, which showed high resistance to chlorantraniliprole.

The LC_50_ for spinosad ranged from 0.0002 to 0.006 µg/ml, and the RRs ranged from 0.02 to 0.50, indicating that all field populations exhibited susceptibility/low resistance to spinosad.

## Discussion

Information on the susceptibility status of insect vectors to commonly used insecticides is a key aspect in the selection of the most appropriate and effective insecticide [13, 38, 39]. In this study, the eight *C. quinquefasciatus* populations showed varying levels of resistance, ranging from low to high, against cyromazine. Cases of resistance to cyromazine have been documented in various medical and veterinary pests around the world. For example, different levels of cyromazine resistance have been found in the house fly *Musca domestica* (RR = 4.8 [40], RR = 62.5 [41], RR = 5.6 [42], RR = 18.0 [43, 44] and RR = 2.9 [13]) and in the Australian sheep blowfly *Lucilia cuprina* (RR = 12.8 [45]).

In the present study, all test populations of *C. quinquefasciatus* exhibited a high level of resistance to the IGR diflubenzuron; in contrast, for the IGR triflumuron, one population showed high resistance, five showed moderate resistance and two showed susceptibility/low resistance. Diflubenzuron has been found to be an effective IGR against the stable fly *Stomoxys calcitrans* and the mosquitoes *Aedes aegypti* and *Aedes albopictus* [23–25]. However, a high level of diflubenzuron resistance was found in *M. domestica* (RR = 120) [40], in the sheep body louse *Bovicola ovis* (RR = 90) [46] and in the mosquito *Culex pipiens* (RR = 128) [47]. Similarly, triflumuron resistance has been well documented in *B. ovis* (RR = 93.8) [46]. Triflumuron has been found found to be an effective IGR against *M. domestica* [13, 44] and against *C. quinquefasciatus* [48].

Susceptibility/low resistance to the avermectin abamectin was detected in the *C. quinquefasciatus* populations in the present study. Previously, resistance to abamectin has been documented in *M. domestica* (RR = 5.9–94.4) [22, 49].

We also detected susceptibility/low resistance to diamide chlorantraniliprole in all *C. quinquefasciatus* populations from the tested regions, with the exception of the IRQ population which showed high resistance (RR = 11.93) when compared with the susceptible strain. Previously, field-evolved resistance to chlorantraniliprole has been reported in *M. domestica* (RR = 36) [28].

In this study, susceptibility/low resistance to the spinosyn spinosad was detected in the *C. quinquefasciatus* populations from the tested regions. Field-evolved resistance to spinosad has been reported in *M. domestica* (RR = 7.2) [22].

The evolution of insecticide resistance arises when a population has been intensively exposed to a pesticide, resulting in the elimination of susceptible individuals and the survival of resistant ones [21]. In this study, susceptibility/low resistance to abamectin, chlorantraniliprole and spinosad is an interesting finding and could could reflect the low or absence of usage of these insecticides against mosquitoes in Saudi Arabia, although insecticides with such novel formulations are commonly used against agricultural insect pests in Saudi Arabia.

This study highlights high levels of resistance in *C. quinquefasciatus* to diflubenzuron and triflumuron, possibly due to: (i) cross-resistance arising from concurrent usage of conventional insecticides like organophosphates and pyrethroids [13]; (ii) target-site mutations or involvement of metabolic enzymes [47, 50–52]; (iii) extensive usage of these insecticides in mosquito control programmes [53]; (iv) detoxification by enzymes [54, 55]; and/or (v) lack of effective and successful resistance management strategies [21]. Resistance to cyromazine is emerging in some *C. quinquefasciatus* populations, so this insecticide should be used rotationally in those locations where resistance has developed to delay further development of resistance. Nevertheless, further studies on cross-resistance patterns, metabolic mechanisms and target-site resistance mutations will confirm the exact phenomena. Laboratory selection of *C. quinquefasciatus* under the influence of diflubenzuron is currently under evaluation to explore cross-resistance and the mechanisms of resistance.

## Conclusions

In conclusion, the evidence from this study of resistance to the IGRs cyromazine, diflubenzuron and triflumuron in *C. quinquefasciatus* indicates the potential lack of systematic management practices in Saudi Arabia. We believe that it is necessary to urgently establish effective resistance management strategies to delay the further development of resistance to these IGRs. Resistance management strategies, including the rotational use of potent insecticides, the integration of cultural practices, such as removal of breeding places, and the use of biological control agents [9], should be applied for the management of *C. quinquefasciatus* to minimise over-reliance on insecticides. The susceptibility/low resistance of *C. quinquefasciatus* larvae to abamectin, chlorantraniliprole, and spinosad suggests that these insecticides retain good potency and hence should be used rotationally with IGRs to sustain their efficacy. Periodic monitoring of resistance to these insecticides should be continued to detect any further increases in resistance. The findings of this study can serve as a reference in future monitoring efforts of *C. quinquefasciatus* insecticide susceptibility.

## Data Availability

The datasets generated and/or analysed during the present study are available from the corresponding author on reasonable request.
